# On-Tree Mango Fruit Size Estimation Using RGB-D Images

**DOI:** 10.3390/s17122738

**Published:** 2017-11-28

**Authors:** Zhenglin Wang, Kerry B. Walsh, Brijesh Verma

**Affiliations:** 1 Centre for Intelligent Systems, Central Queensland University, Rockhampton, Queensland 4701, Australia; z.wang@cqu.edu.au (Z.W.); b.verma@cqu.edu.au (B.V.); 2 Institute for Future Farming Systems, Central Queensland University, Rockhampton, Queensland 4701, Australia

**Keywords:** allometry, fruit size, RGB-D camera, machine vision, precision fruiticulture, time of flight

## Abstract

In-field mango fruit sizing is useful for estimation of fruit maturation and size distribution, informing the decision to harvest, harvest resourcing (e.g., tray insert sizes), and marketing. In-field machine vision imaging has been used for fruit count, but assessment of fruit size from images also requires estimation of camera-to-fruit distance. Low cost examples of three technologies for assessment of camera to fruit distance were assessed: a RGB-D (depth) camera, a stereo vision camera and a Time of Flight (ToF) laser rangefinder. The RGB-D camera was recommended on cost and performance, although it functioned poorly in direct sunlight. The RGB-D camera was calibrated, and depth information matched to the RGB image. To detect fruit, a cascade detection with histogram of oriented gradients (HOG) feature was used, then Otsu’s method, followed by color thresholding was applied in the CIE L*a*b* color space to remove background objects (leaves, branches etc.). A one-dimensional (1D) filter was developed to remove the fruit pedicles, and an ellipse fitting method employed to identify well-separated fruit. Finally, fruit lineal dimensions were calculated using the RGB-D depth information, fruit image size and the thin lens formula. A Root Mean Square Error (RMSE) = 4.9 and 4.3 mm was achieved for estimated fruit length and width, respectively, relative to manual measurement, for which repeated human measures were characterized by a standard deviation of 1.2 mm. In conclusion, the RGB-D method for rapid in-field mango fruit size estimation is practical in terms of cost and ease of use, but cannot be used in direct intense sunshine. We believe this work represents the first practical implementation of machine vision fruit sizing in field, with practicality gauged in terms of cost and simplicity of operation.

## 1. Introduction

On-tree estimation of fruit size is useful for the prediction of maturity and harvest time [1,2], and estimation of crop yield [1,3], and can inform packing material (tray insert) purchasing and marketing arrangements [4]. For physiological studies, measurement of the size of individual fruit over time allows estimation of fruit expansion rate and its response to disease and agronomic conditions [3,5,6,7]. Fruit size estimation is also relevant to autonomous harvesting, for the control of the harvest arm approach and grasp of the fruit.

Mango fruit size varies between varieties, but a typical variety size variation from stone hardening to harvest maturity is 60 to 160 mm in length, 50 to 130 mm in width, with a rate of change of 0.3 and 0.2 mm/day in length and width respectively over this period [8]. In Australia, uniformly sized fruits are typically packed into a 7 kg tray, with tray sizes that range between 10 (average 700 g fruit) and 22 fruits per tray (average 320 g fruit). With each step in tray size, average fruit mass steps by between 40 and 70 g, and fruit length between 4 and 10 mm [9].

Mango fruit mass (*M*, in g) can be estimated from the product of the lineal dimensions (in cm) of fruit length (*L*), width (*W*) and thickness (*T*) [10]. Anderson et al. [8] demonstrated that this relationship is robust across growing conditions (e.g., water stress) and growth stage, with a typical relationship of M=kLWT, where *k* is a constant that varies from 0.49–0.51 (R^2^ = 0.97, RMSE = 28.7 g). However, in viewing fruit on canopy, while *L* can be assessed, there is no control of the orientation of the fruit such that the width of the fruit in the image will range from *W* to *T*. The allometric relationship between lineal dimensions and fruit weight can be further simplified to: $M=kL{\left(\frac{W\;+\;T}{2}\right)}^{2}$ (R^2^ = 0.93) [8].

Mango size estimation has been achieved in context of pack-line fruit grading. Computer vision-based mango size estimation methods have been proposed, based on the use of single [11] or multiple [12] calibrated colour cameras. In these applications, background colour can be chosen to maximise contrast to the fruit, camera-to-holder distance is known, and fruit thickness can be estimated from side views of the fruit.

Lineal dimensions of fruit on the tree can be assessed manually using Vernier callipers or sizing rings. Such measurements are labour-intensive and tedious, especially in the hot, humid field environment of the mango fruiting season. A machine vision system could be mounted to farm vehicles to provide non-contact measurement of fruit size. There has been an explosion in the use of machine vision in fruit recognition and estimation of fruit count on tree (e.g., see reviews by Goongal et al. [13] and Payne et al. [14]), and emerging machine learning technologies have achieved remarkable performance in the recognition and localization of fruit for the estimation of fruit number on tree [15,16,17,18]. However, to use machine vision for fruit sizing, an un-occluded view of the complete contour of the fruit and the camera-to-fruit distance is required. Further, while estimation of a sample of fruit is sufficient to inform an estimate of fruit size distribution, the caveat applies that the fruit visible on the outer canopy are representative of all fruit on the tree or at least of fruit to be harvested.

For estimation of mango fruit size on tree using machine vision, fruit detection, object size in pixels and camera to fruit distance are required. However, while it is required that ‘intact’ (non-occluded) fruit be assessed, only a statistically relevant number of fruit need be assessed, not all fruit (as is required for estimate of crop load).

For fruit detection, we have previously reported on the use of dense texture-based segmentation algorithm, a color and elliptical shape based model and a simple color and smoothness filter based method [15,18,19]. More recently, deep learning technology (Faster Regional Convolutional Neural Network) has been applied to mango fruit detection [16,17]. However, as the high computational cost of deep learning currently hampers its application for real-time tasks such as in-field fruit sizing, feature-based methods remain of interest. For example, mango fruit has the distinguishing feature of an arc edge, suited to rapid characterization using Histogram of Oriented Gradients (HOG) [20] features. In the current study we propose to continue the use of color thresholding and ellipse fitting, but with addition of cascade detection with HOG features instead of Support Vector Machines (SVM) with Scale-Invariant Feature Transform (SIFT) features.

For estimation of mango fruit size on tree, an accurate estimate of fruit lineal dimensions in image pixels must be converted to actual size (mm) using the thin lens theory. To achieve this, either a scale is required in the plane of the fruit, which is impractical, or the distance of the camera to the fruit is required. A summary of relevant approaches follows:

- Reference scale: A reference object with known size may be included in the image, allowing for the estimation of size of target objects by referring to the reference scale. This method is simple but requires the reference object to be placed on the same plane as the target, which makes it impractical. For example, Cheng et al. [21] utilised 50 mm diameter white and red spheres placed on trees in the estimation of apple sizes in an orchard, with accurate estimation of size only for the fruit close to the reference objects.
- Ultrasonics: Murali and Won Suk [22] used ultrasonic sensors and machine vision techniques to estimate citrus fruit size on trees. This work employed four ultrasonic sensors to estimate an average distance between the tree and the camera for calculation of fruit size, and the maximum one of estimated diameters of fruit in images was used to represent the fruit size. An unacceptably large RMSE = 19 mm was obtained, with error ascribed ultrasonic depth inaccuracy, clustering of fruits, and variable illumination.
- Stereo or multi-view imaging: in stereo imaging, spatial displacement of images from a pair of cameras is converted to real distance by triangulation. Although many algorithms have been proposed, evaluating stereo imaging depth still faces difficulties in matching correspondence points due to occlusion, similarity, variation in light levels, imaging noise and calibration errors [23,24,25]. Application to fruit within a mango canopy faces even more difficulties, as complex canopy geometry can cause failures in finding stereo pairs [16]. For example, Font et al. [26] proposed a stereovision system for automated fruit harvesting with reported measurement errors of up to 76 mm in distance estimation at a camera-to-fruit distance of 2025 mm and up to 5.9 mm in fruit diameter estimation.
- Laser rangefinder: laser distance and LiDAR units emit pulsed laser beams, with measurement of the round-trip Time of Flight (ToF) of light offering precise distance information [27,28]. A ToF point distance measurement of a specific fruit could be associated with the camera image of that fruit. LiDAR allows for whole canopy imaging and are insensitive to sunlight, but spatial resolution at on-the-go speed of movement is low, in terms of localization of fruit. LiDAR units are also effectively an order of magnitude higher in price than the other sensors under consideration. Single point laser rangefinders offer a cost-effective access to ToF technology.
- ToF-based RGB-D camera: ToF cameras assess distance to all image pixel points by emission of a cone of modulated continuous light-wave and measurement of the phase shift of the received light-wave to obtain the travel time of light [28,29]. In this kind of camera, each detector pixel performs demodulation of the received signal independently, and therefore the camera is capable of measuring the depth from a whole field of view simultaneously. Post-image processing techniques can correspond depth and RGB information at a pixel level, although RGB-D cameras typically have a relatively low spatial resolution. Several commercial RGB-D cameras are available on the market. The low cost Microsoft Kinect V1 RGB-D camera was used for plant phenotyping by Paulus et al. [30] and apple fruit localization within a tree canopy [31], and the Kinect V2 camera was used in measure of structural parameters of cauliflower by Andújar et al. [32].

In the current study, we extend our previous work employing a farm vehicle-mounted machine vision system with Light Emitting Diode (LED) illumination for mango fruit count estimation [15,18], with improvement in the machine vision algorithm used for detection of fruit and addition of camera to fruit distance measurement to allow for fruit sizing. The practical goal of this work is a system for mounting to farm machinery, operating at a speed of approximately 5 km/h, able to estimate length and width of a sample of fruit to within 4 mm, allowing estimation of fruit weight to within 40 g. As in the previous work, imaging was undertaken at night to achieve consistent illumination, thereby facilitating image processing. Other researchers have also reported that night imaging can significantly improve fruit detection accuracy [33]. Further, a number of farm management activities are undertaken at night in the Australian mango industry (e.g., spraying), including a trend to night harvesting, so night imaging can fit within existing practices.

For the estimation of the size distribution of fruit in an orchard, high precision (i.e., zero false positive errors) is desired in detection of un-occluded fruit but false negative errors (missing detection of fruit) are not an issue. We extend our previous work on fruit detection [15,18,19] through the use of cascade detection with HOG features [34]. For estimation of camera-to-fruit distance, low-cost examples of three distance measurement technologies were compared, (i) a Zed stereo vision camera, (ii) a Leica handheld ToF distance meter and (iii) a Kinect ToF RGB-D camera. Accuracy of ToF distance measurement was also considered in context of fruit, which act as diffuse rather than specular reflectors, and in the context of operation in sunlight.

## 2. Materials and Methods 

### 2.1. Distance Measurement Technology—A Description

Three low cost distance measurement technologies were compared for use in a fruit sizing system, to be mounted on a moving platform equipped with LED illumination for night imaging of mango orchards. The imaging platform employed a number of sensors surrounded by LED flood lights, expanding the orchard imaging system described by [18]. LED light output was in the wavelength range from 450 to 750 nm. Sensors included a Leica laser distance meter (DISTO D2a) positioned directly above the depth sensor of a Kinect V2.0 camera and adjacent to a Zed stereo camera.

The Zed stereo camera (https://www.stereolabs.com, cost < US$500) consists of two wide-angle lenses with a resolution of 3840 × 1080 pixels at 30 fps, an f-stop of 2, and a baseline distance of 120 mm. Each lens captures a slightly different view of the target scene. By comparing the displacement of pixels between the left and right views, the depth of the scene was estimated through triangulation using the software provided by the camera vendor. The Zed camera can work in a depth range of 0.5–20 m. Compared with other technologies, stereo depth imaging is a passive approach without requirement of additional light source, such that it can be used in both indoor and outdoor environments.

The DISTO D2a (Leica, Heerbrugg, Switzerland, cost < US$500) is a handheld laser distance meter with a claimed accuracy of ±1.5 mm and depth range up to 100 m. It emits only one pulsed light beam, so that it measures depth information to one point at one time. The high laser intensity and narrow bandwidth filter used to reduce the interference from ambient light enables the use of indoor and outdoor in full sunlight. The distance meter has an inbuilt red laser pointer to mark the point of distance measurement. As an imaging platform moves down orchard inter rows, the point traverses the canopy, sometimes traversing un-occluded fruit. These fruits can be identified in the image, with the distance value used in calculation of fruit dimensions from the RGB image.

The Kinect RGB-D cameras (Microsoft, Redmond, WA, USA) were developed to gather distance images for gaming environments, and they market for <US$200. The first generation of these units projected a structured light pattern, the distortions of which were used in the estimation of distance. The second generation uses a ToF technique, with a time delay on imaging (512 × 424 pixels) of a modulated 820–860 nm light wave used in estimation of depth. The ToF image is matched to the RGB image of a second camera (1920 × 1080 pixels). The system has been characterized by Lachat et al. [35] and Pagliari and Pinto [36], with a residual error on depth measurement of <20 mm (for distances from 0.5 to 4 m) reported. The working range of 0.5 to 4.5 m is set by the modulation frequency of the illuminating light. The camera measures the vertical distance between the camera plane and the object plane rather than the straight distance from the camera to the object. The RGB camera requires an external illumination source, but the ToF camera is reported to suffer interference from sources containing wavelengths near 850 nm (e.g., sunlight) with standard deviation on repeated measures of a target at 1.2 m distance reported to increase to 35 mm in full sunlight [37,38].

### 2.2. Distance Measurement Accuracy and Precision

Depth accuracy and precision affects the estimation of fruit size. For instance, at a camera to object distance of 2 m, an error of 10 mm in distance measurement will cause an error of 0.5 mm on the estimation of a 100 mm long object.

Distance measurement was undertaken under laboratory conditions (fluorescent lighting) using three materials varying in levels of diffuse reflectance (ceramic tile, Polytetrafluoroethylene (PTFE) block, and fruit). Target objects were placed at 14 positions ranging from 0.56 to 5.4 m (device to object distances, measured with a metal tape with 1 mm markings), with the object surface parallel to the device plane. The Leica distance meter and Zed camera were mounted to the top of the Kinect camera and aligned with the front surface of the Kinect camera. The ceramic tile to Kinect distance measurement exercise was repeated outdoors at times from early afternoon to after sunset (14:30 to 17:00 h).

### 2.3. RGB-D Image Calibration and Registration

Within the Kinect device, the RGB and depth camera lenses and sensor arrays are separated horizontally by approximately 55 mm. This separation can incur translation, rotation, and shearing of images. The calibration procedures reported by [35,36] were adapted to map objects of interest in images from both cameras to the universal world coordinate system.

Kinect infrared (grayscale) and RGB images (*n* = 20) were captured of an 8 by 9 grid checkerboard with a grid side length of 34 mm, with the position of the board varied from 1.5 to 2.5 m from the cameras. This distance range is similar to that of fruit imaging from inter-rows in an orchard. MATLAB (MathWorks, Natick, MA, USA) calibration tools were adopted with the default calibration configuration, i.e., skew and tangential distortion were not considered, and only two radial distortion coefficients (K1 and K2) were used. The estimated calibration parameters (Table 1) were then used to rectify the color and depth images. The extrinsic matrix consisted of a 3-by-3 rotation operation, and a translation vector (the fourth column vector), which was used to map depth pixels with color pixels following the procedure in [39]. As the Kinect RGB camera has a wider field of angle in the horizontal direction and a smaller field of angle in vertical direction than its depth camera, not all of the RGB pixels can be matched with depth information. The RGB image must therefore be cropped to match the depth image. The RGB camera sensor size and focal lengths of *x*, *y*-axis (Table 1) were used to calculate the fruit lineal dimensions according to the thin lens theorem.

### 2.4. In Orchard Activity

Field work was conducted at night (18:30~21:30 h) on two occasions, with fruit at two stages of maturity (pre- and post- stone hardening). Fruit that were visually well-separated in each of 25 canopy images were identified and measured manually using calipers. Five randomly selected images were used in tuning the parameters of the proposed algorithm and training for cascade fruit detection, and the remaining images were used in validation.

In another exercise, the position and weight of all fruit on one tree was assessed. A plumb bob on a string was held at the base of each fruit. The bob position was recorded relative to tree trunk center using two pieces of (2 cm-sided) square cross section steel tubing. One tube was placed along the tree row, while the second was placed perpendicular to the first tube, and moved along it until positioned under the plumb bob for each fruit. A metal tape measure was used in assessment of *x*, *y* and *z* distances. All fruit (*n* = 136) were numbered while on tree, then weighed individually after harvest.

### 2.5. Cascade Fruit Detection

**Figure 1:**
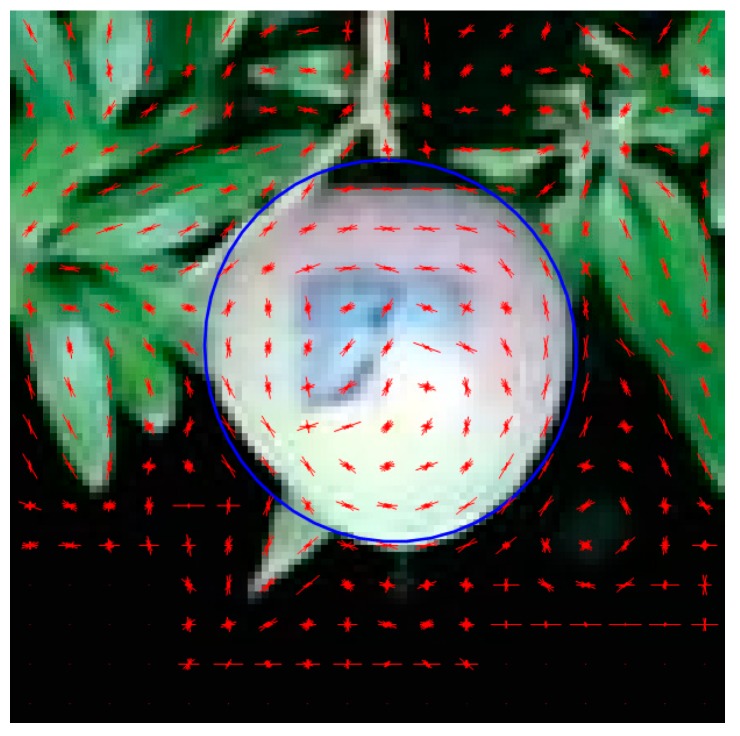
Histogram of Oriented Gradients (HOG) feature representation of mango fruit.

MATLAB was used to implement the proposed approach. A cascade classifier with HOG features was trained based on hand-crafted fruit image snips. A box size of 6 × 6 and cell size of 6 × 6 pixels was selected for HOG feature extraction (Figure 1). As this study aims at estimation of fruit size rather than count, zero false positive error (negative samples incorrectly classified as positive samples) was required. A relatively strict merge threshold of 8 was set for the cascade classifier to reduce false positives. However, the cascade fruit detection result cannot be used directly for fruit size estimation as (i) there can be false positives; (ii) the output bounding box does not tightly encapsulate the complete fruit; (iii) detected fruit that are partially occluded should be excluded from size estimation.

### 2.6. Pixel Based Segmentation for Background Removal

To address the above issues, pixel-based segmentation was conducted on the image snips from the cascade fruit detection. The original bounding box containing detected fruit was doubled in size to ensure coverage of the entire fruit. The image snip was then converted into CIE L*a*b* color space and Otsu’s method [40] was used to separate the fruit contour in the L* channel. This unsupervised approach calculates the optimum threshold separating the image into two classes of pixels, so that the intra-class variance is minimal, creating a binary image. Because of their convexity and smoothness, mango fruit generally reflect more light than surrounding leaves or branches in illuminated night images, such that this method can separate fruit from the background [41]. Remaining leaf and branch related pixels were removed using a fixed color threshold based on a* and b* channels (−25 ≤ a* ≤ 25, −20 ≤ b* ≤ 35), and a morphological operation of area opening was applied to remove small objects with area <300 pixels, followed by closing to fill holes of <300 pixels in fruits. Pixels of non-interest were assigned zero values.

### 2.7. Stalk Removal

The presence of the mango peduncle (fruit stalk) in an image can result in failure to differentiate the true fruit contour. As the fruit stalks are much thinner than fruit, a line-based 1D filter was developed to remove these features from images. If the number of non-zero pixels between two zero-values was less than a predefined number w, the non-zero pixels were removed (set to zero). The filter was performed line by line horizontally and then vertically. Horizontal filtering can remove stalks connecting to fruit, with the weight of fruit ensuring that the stalks hang vertically. Vertical filtering can remove those stalks or leaves touching the side of fruit. Because the filter was executed on individual lines in both horizontal and vertical directions, fruit shape was generally maintained. By contrast, common morphological filters performed in all directions can cause the filtered object to lose its original shape. The value w=8, roughly equivalent to 14 mm at a camera to object distance of 2 m, was empirically selected. However, the filter truncated the end of imaged fruit with a ‘sharp’ tip. Therefore, a dilation operation using a disk shape with radius of 1 pixel was used in compensation.

### 2.8. Ellipse Fitting for Recognition of Complete Fruit

**Figure 2:**
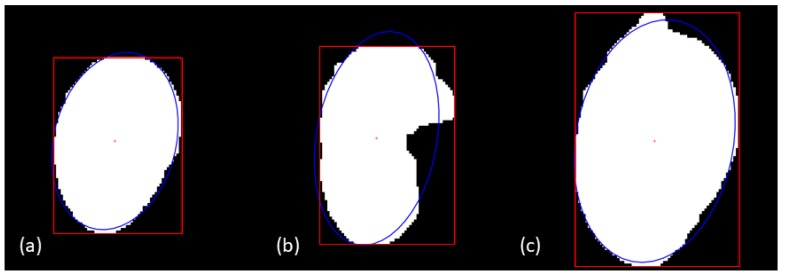
Ellipse fitting: (**a**) An identified well-separated fruit with ϵ=0.65 and r=0.99; (**b**) failure to fit an ellipse as r=0.93; (**c**) failure to fit an ellipse as bounding box (red) length is 4 pixels greater than ellipse (blue) major axis.

The above detection routines included fruit with incomplete contours, i.e., partly occluded by a leaf or another fruit. As mango fruit has an approximate ellipse contour, a simple method of ellipse fitting based on image moment [42] was used to identify if the imaged fruit has a complete contour. This method of measuring ellipticity has been reported to yield better accuracy than other methods [43,44,45], and to have a good error tolerance (i.e., tolerance of imperfect ellipse shapes, as for mango fruit). Four criteria were used to judge whether the fruit contour was complete:

1. Ellipse area (A): a whole fruit at a camera distance of around 2 m should have an area of 1000 to 8000 pixels. Smaller patches could be background or incomplete fruit, while larger patches could be clustered fruit.
2. Area ratio (r): the ratio of real connected component (a segmentation result of a fruit) size (A) to calculated ellipse area based on the fitted ellipse major axis a and minor axis b, defined by:
  (1) $$r=\frac{4A}{\pi ab}$$
  The value of *r* should be >0.97 if a whole mango is imaged, with the mango filling the detection ellipse fully, while stalks or leaves do not.
3. Eccentricity (ϵ): the ellipse encapsulating a mango fruit is closer to a circle, leading to a relatively small ϵ than values for non-mango objects, such as leaves or fruit clusters. A connected component with ϵ>0.75 was rejected.
4. Bounding box length versus ellipse major length: the length of a bounding box just encapsulating the fruit was used in estimation of the fruit length. The major axis of the fitted ellipse is usually larger than the length of the bounding box (Figure 2), however, thick stalk ends that were not removed by the line filter could result in an overestimation of fruit length. Therefore, if the length of bounding box was 4 pixels larger than the ellipse major axis, the object was excluded from size estimation.

### 2.9. Fruit Size Estimation

Once a connected component was recognized as a complete fruit, a bounding box was created, being the smallest rectangle containing the component. The RGB image pixel associated with the centre of the bounding box was registered to the associated depth image. The average of a 2 × 2 pixel array about the registered point was accepted as the camera-to-object distance (D). Fruit with a depth value less than 0.8 m were excluded from size estimation. The real length and width of the fruit was calculated according to the thin lens theory:

(2) $$\frac{f}{D}=\frac{\mathrm{image}\;\mathrm{size}}{\mathrm{real}\;\mathrm{size}}$$

where f is focal length, D is the fruit to camera distance obtained from RGB-D camera depth information, and image size is obtained from the product of the number of fruit pixels and sensor size. The x- and y-axis focal lengths of the RGB camera were different (see calibration results in Table 1), and were used to calculate fruit length and width respectively.

### 2.10. Workflow of Proposed Method

**Figure 3:**
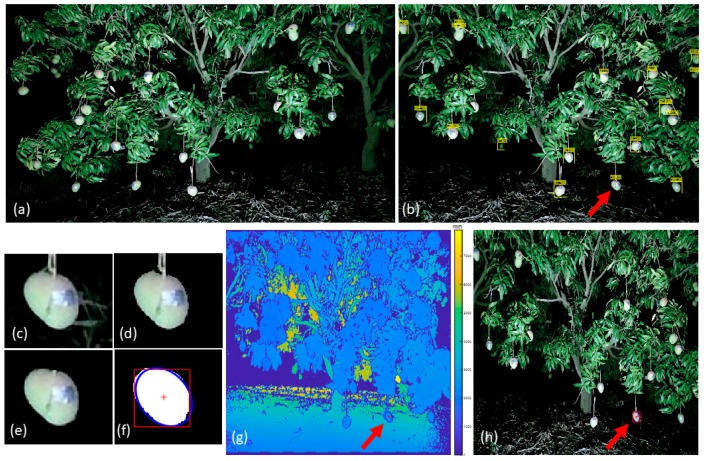
Fruit detection method: (**a**) original input Kinect RGB image; (**b**) cascade fruit detection using HOG features; (**c**) an identified fruit by cascade fruit detection; (**d**) background removal with Otsu’s method and color thresholding; (**e**) stalk filtering; (**f**) and (**g**) depth registration; (**h**) fruit size calculated from bounding box side lengths; with (**g**) displaying the point of depth registration on a single fruit and its bounding box. The red arrow points to the same fruit in panels **b**, **g** and **h**, and this fruit is shown in panels **c**–**f**.

The image processing method consisted of the following steps (Figure 3): (i) Kinect RGB images were cropped to match the field of the depth image (Figure 3a); (ii) the raw image was mirrored on the vertical axis (the Kinect image is presented for gaming use), and fruit detection was undertaken based on a cascade detection with HOG feature (Figure 3b,c); (iii) background was removed using Otsu’s method based on L* values, followed by color thresholding based on a* and b* (Figure 3d); (iv) fruit peduncle was removed using a line filter (Figure 3e); (v) un-occluded fruit were detected based on moment-based ellipse fitting [42]; (vi) camera-to-fruit distance was obtained (Figure 3f,g); (vii) a bounding box was used in estimation of fruit length and width (in pixels) (Figure 3f,h); and (vii) fruit lineal dimensions were calculated using the thin lens formula.

## 3. Results

### 3.1. Distance Sensor Comparison

**Figure 4:**
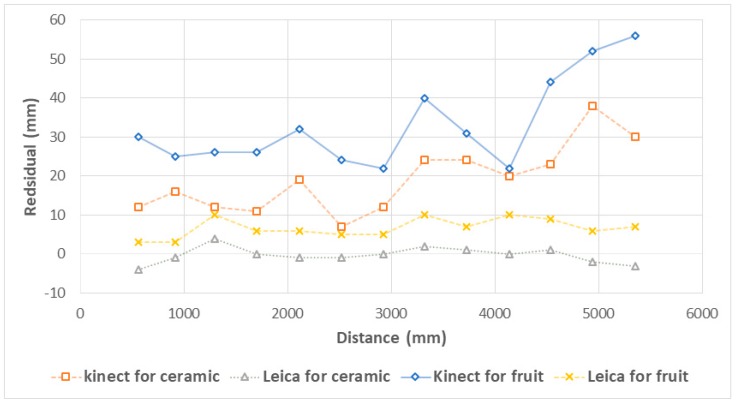
Plot of residual of measured to actual distance from device to object as a function of distance to a ceramic tile and to a fruit for Kinect and DISTO technologies (Zed camera results could not be shown on this y scale). Solid lines are used for ceramic measurements, dotted lines for fruit.

The Zed stereo depth imaging technique was inferior to other technologies, achieving a bias-corrected RMSE of 126.9 and 155.6 mm on ceramic tiles and fruit, respectively (Table 2). The Leica laser distance meter achieved a bias-corrected RMSE of 2.0 and 2.4 mm on distance to a ceramic tile and fruit, respectively (Table 2), with nearly equal performance at all distances between 0.6~5.4 m (Figure 4). The Kinect RGB-D achieved a bias-corrected RMSE of 8.4 and 11.0 mm on distance to a ceramic tile and fruit, respectively, consistent with the precision reported in [36]. Error increased at distances >4.5 m (Figure 4), consistent with the specification for use over the range 0.5–4.5 m. The increase in bias between ceramic and PFTE or fruit targets was larger for the RGB-D device (12.0 mm) than for the laser distance device (4.8 mm). For fruit, a bias corrected RMSE of 11 mm was achieved with the RGB-D device.

In direct sunlight with a ceramic target, the RGB-D camera failed to measure distance information for distances over 3.5 m. For the range 0.6 to 3.5 m, an RMSE of 14.6, 10.5, 9.4 and 8.4 mm was achieved at 14:30, 16:00, 17:30 and 19:00 h, respectively (with sunset at around 18:00 h).

### 3.2. Fruit Detection

The parameters for color thresholding and ellipse fitting were explored using training set images and then applied to the validation set. Strict ellipse criteria (r>0.97, ϵ<0.75) were required to avoided false positives in fruit detection, although this was at the expense of total positive detections.

The cascade fruit detection method identified 353 objects in the validation images, but with false positives (precision = 81%, Table 3). Applied to the output of the cascade fruit detection, the ellipse fitting method decreased the total object count (*n* = 90, 77% of a human assessment of non-occluded fruit in the image set) but eliminated false positives and occluded fruit (Table 3).

### 3.3. Estimation of Fruit Dimensions

Across all fruit assessed using manual calipers, average fruit length/width/thickness was 101, 81, 73 mm, respectively. The minimum-maximum fruit length, width and thickness were 56–149 mm, 47–123 mm and 42–101 mm. The Standard Deviation (SD) on repeated digital caliper measures of fruit lineal dimension by the same operator was 1.2 mm.

**Figure 5:**
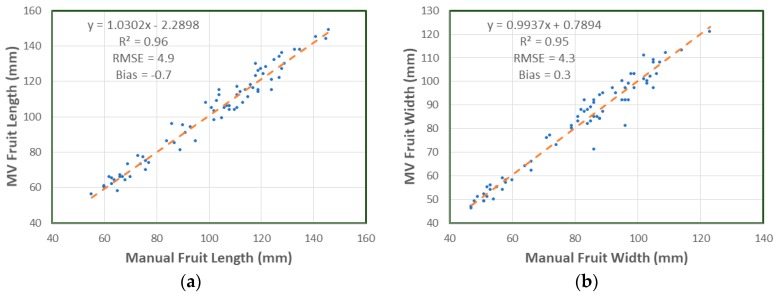
Machine vision (MV) and manual caliper measurement of: (**a**) length; (**b**) width of mango fruit on tree for the validation set images.

While the camera view of fruit on tree allowed estimation of *L*, it provided images of fruit in orientations that resulted in estimation of a width value that ranged from *W* to *T*. Therefore, the Machine Vision (MV) estimate of fruit width was compared to the closer value of manually assessed *W* and *T*. The correlation between manual measurement and the machine vision based estimate was characterized by R^2^ = 0.96 and RMSE = 4.9 mm for fruit length estimation and R^2^ = 0.95 and RMSE = 4.3 mm for fruit width estimation (Figure 5). The bias of −0.7 mm on length and 0.3 mm on width was within the uncertainty of the reference measurement (SD = 1.2 mm).

For fruit mass estimated using an allometric relationship with fruit lineal dimensions (Mass = 0.42 × L × W^2^) [8], the assessed measurement error (4.9 mm for length and 4.3 mm for width) would result in an error of 45 g (calculated for the average fruit assessed, i.e., 101 mm length, 81 mm width, weight 278 g).

### 3.4. Sampling Considerations

**Figure 6:**
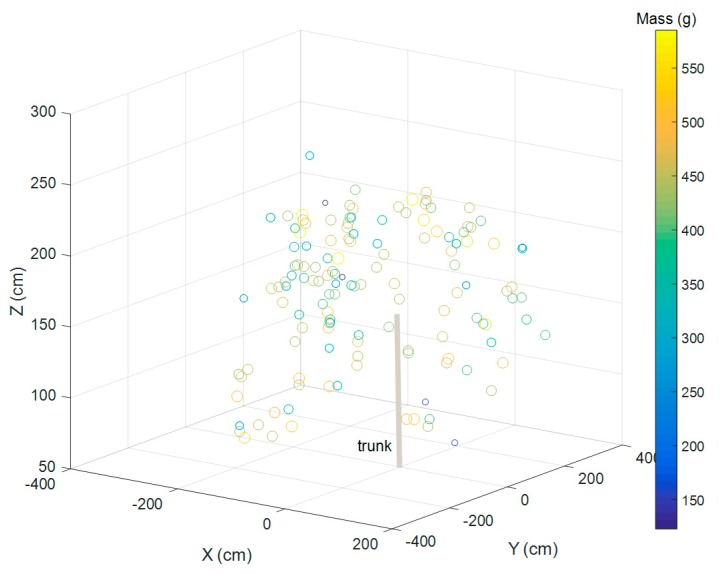
Representation of fruit position within a tree canopy, colored and sized by fruit mass. The **X** axis is perpendicular to the orchard row, the **Y** axis is parallel to the orchard row, and the **Z** axis is tree height. The co-ordinate (0, 0, 0) represents the center of the trunk at ground level. The scale bar represents fruit mass (150 to 550 g).

All fruit on one tree were physically located in three dimensions and each fruit was weighed at harvest. There was a negligible gradient in fruit mass between outer and inner canopy (Table 4, Figure 6).

## 4. Discussion

### 4.1. Choice of Distance Sensor

For an object of 100 mm length measured at 2 m distance, camera to fruit distance errors (bias corrected RMSE) of 155.6 mm (stereo imaging), 2.4 mm (laser distance) and 11.0 mm (RGB-D) will result in ±7.8 mm, ±0.12 mm and ±0.55 mm errors in estimation of object length, respectively (Equation (2)). Therefore, the Zed stereo depth imaging system is not suitable for the application of on-tree fruit sizing.

Once determined, bias can be corrected in a measurement system. The bias on front of camera to ceramic tile distance measurement for the RGB-D device was 19.1 mm, a result attributed to the distance between the outer surface of the device (measurement point) and the sensor within the Kinect device. The bias on the laser distance measurement was negligible (−0.3 mm).

It has been previously reported that the distance measurements of the Kinect (V2) device is impacted by the translucency of the object material [46]. The increase in bias (of approximately 10 mm) for both laser distance and RGB-D devices with assessment of PFTE and fruit is consistent with light scattering within these semi-translucent media, resulting in an increase in the average time of flight of photons returning to the sensor. This phenomenon is utilized in time and spatially resolved spectroscopy of fruit to separate absorption and scattering coefficients [47].

The use of the handheld laser distance meters involved detection of the measurement point on an un-occluded fruit on the tree canopy. With the current arrangement of a single RGB image acquisition per tree from the mobile imaging rig, this approach results in the measurement of fewer fruit than with use of the Kinect sensor, as it requires the coincidence of the measurement spot and fruit in the image.

A potential limitation of the RGB-D camera is its operating range of 0.5~4.5 meters. However, this range suited the current application, with typical camera to fruit distances of 1.0 to 3.0 m. Therefore, we focused attention to use of the RGB-D camera.

### 4.2. Fruit Detection

The proposed fruit detection method was chosen for detection of un-occluded fruit, rather than the detection of all fruit in the image, in context of estimation of fruit dimensions. The proposed method of combining cascade detection and ellipse fitting demonstrated high precision but poor detection rate, a result suitable for this application. Of 353 fruits identified by cascade fruit detection, 90 fruits were identified as un-occluded by leaves, branches or other fruit, with an average of 4.5 fruits per tree (image). The applicability of the ellipse fitting technique for fruit detection remains to be trialed with fruit of a range of cultivars, varying in fruit shape.

### 4.3. Estimation of Fruit Dimensions

Mango fruit are not perfectly elliptical, causing error in the estimate of fruit length and width from the ellipse axis lengths. The bounding box method was more accurate than the ellipse method and was therefore implemented.

Fruit segmentation was a source of error in the sizing estimation. The Kinect RGB camera has a small focal length lens of approximate 3.5 mm and coarse resolution sensors of 1920 × 1080 pixels with a pixel side length of 3.1 μm. At a camera to fruit distance of 2.0 m, the typical mango fruit has an image of approximately 70 pixels by 50 pixels. A one-pixel error on the segmentation of a fruit edge will introduce an error of $\left(3.1\times 10^{-3}\times 2000÷3.5157\right)\approx 1.76$ mm on estimation of length or width. Segmentation error could be reduced by introducing edge detection techniques such as Canny edge detection and key-point detection [48]. Fruit depth profile information from the RGB-D camera was not of practical use in fruit segmentation due to the lack of spatial resolution (RGB-D depth images being 512 × 424 pixels).

A higher resolution RGB camera with greater focal length could be used to improve lineal measurement accuracy. For example, for the Canon 50D DSLR camera (4752 × 3168 pixels; pixel size of 4.99 μm) with a 28 mm wide angle lens used in previous work [18], one-pixel segmentation error results in only 0.35 mm error in estimation of fruit width or length. However, the registration error between Kinect depth camera and Canon RGB camera must be taken into consideration.

### 4.4. Sampling Considerations

The proposed machine vision method involves size estimation of only a sample of fruit on each tree, being non-occluded fruit visible in an image of the tree from the inter-row. This method will involve detection of outer canopy fruit only. Inner canopy fruit tend to be smaller than outer canopy fruit, although this difference was not significant in the fruit assessed (Figure 6). This issue represents a potential limitation of the method, where fruit size varies by canopy position.

For the population of fruit used in this study, the Standard Deviation (SD) of manually measured fruit lengths was 24.4 mm. Thus, the minimum number of fruit that should be sampled for an estimate of mean fruit size is 95 (with a 95% confidence level, to an error of 4.9 mm) according to:

(3) $$n={\left(\frac{1.96\times SD}{e}\right)}^{2}$$

where 1.96 is the t statistic for a 95% confidence interval, *SD* is the standard deviation of the population and e is the accepted error (RMSE on measurement). This number of fruit can be easily sampled within an orchard using the proposed machine vision approach, with potential for mapping of spatial variation in fruit size across an orchard.

### 4.5. Implementation

Fruit position and size information can be used in monitoring of fruit maturation, estimation of size class distribution for ordering of packing materials and marketing purposes, and could support selective robotic fruit harvest [13].

The proposed system is simple but practical. Imaging was conducted at night, with the illumination system enabling normal RGB camera exposure times of 1/200 s, avoiding motion blur. Night imaging avoided issues of interference from ambient sun light for the low-cost RGB-D camera employed and simplified object (fruit) detection. The design goal of a system able to be mounted to a farm vehicle operating at 5 km/h was met. The RMSE on fruit length measurement of 4.9 mm was too high to allow classification of smaller fruit to the correct tray size (design criterion was 4 mm accuracy), but adequate for monitoring crop maturation sizing and for sizing to within one tray size tolerance.

## 5. Conclusions

A machine vision method based on low-cost componentry was developed to estimate the size of fruit on tree from an inter row distance of approximately 2 m, with performance of an RMSE of 4.9 mm on caliper measurements of fruit length and 4.3 mm on fruit width. This performance is sufficient for estimation of fruit growth with repeated measures, adequate to assign fruit to within one tray insert size. As a relatively modest number of samples are required to represent the size distribution of fruit in the orchard, the method does not require detection of every fruit on the tree. To improve lineal measurement accuracy, a higher resolution RGB-D camera with greater focal length lens could be used. The system was designed around night imaging, but could also be used in day time hours, during periods of diffuse illumination (dawn, dusk, cloudy conditions).

## Figures and Tables

**Table 1:** Kinect camera intrinsic parameters: the resolution and sensor size information from [36], other parameters obtained via calibration.

	RGB Camera	ToF Camera
Resolution (pixels)	1920 × 1080	512 × 424
Sensor size (μm)	3.1	10
Focal length *x*-axis (mm)	3.2813	3.6413
Focal length *y*-axis (mm)	3.5157	3.9029
Principal Point *x* (pixel)	965.112	263.852
Principal Point *y* (pixel)	583.268	225.717
K1 of Radial distortion	9.3792 × 10^−5^	9.7968 × 10^−5^
K2 of Radial distortion	−7.5342 × 10^−8^	−1.9084 × 10^−7^

**Table 2:** Measurement of object distance using three low cost technologies for different materials over the range 0.6–5.4 m. RMSE-bc refers to bias-corrected root mean square errors (mm).

Criteria	Ceramic			PFTE			Fruit		
	Zed	Leica	Kinect	Zed	Leica	Kinect	Zed	Leica	Kinect
R^2^	0.998	1.000	1.000	0.998	1.000	1.000	0.997	1.000	1.000
Slope	0.929	0.999	1.004	0.907	0.999	1.004	0.923	1.000	1.005
Bias (mm)	−69.3	−0.3	19.1	−62.8	4.5	31.1	−38.7	6.7	33.1
RMSE-bc (mm)	126.9	2.0	8.4	159.5	2.2	8.2	155.6	2.4	11.0

**Table 3:** Fruit detection using cascade fruit detection and cascade detection followed by ellipse fitting methods for validation set images.

	Total Detection	True Positives	False Positives	Precision (%)
Cascade detection	435	353	82	81.1
Ellipse fitting	90	90	0	100

**Table 4:** Fruit mass at different canopy positions.

Position	n	Mean (g)	SD (g)	Max. (g)	Min. (g)
Outer canopy	72	433	82.9	585	123
In between	31	421	75.8	560	222
Inside canopy	33	391	97.1	567	134
Overall	136	420	86.2	585	123
